# Murine Typhus and Febrile Illness, Nepal

**DOI:** 10.3201/eid1410.080236

**Published:** 2008-10

**Authors:** Mark D. Zimmerman, David R. Murdoch, Patrick J. Rozmajzl, Buddha Basnyat, Christopher W. Woods, Allen L. Richards, Ram Hari Belbase, David A. Hammer, Trevor P. Anderson, L. Barth Reller

**Affiliations:** Patan Hospital, Kathmandu, Nepal (M.D. Zimmerman, B. Basnyat, R.H. Belbase); University of Otago, Christchurch, New Zealand (D.R. Murdoch); Canterbury Health Laboratories, Christchurch (D.R. Murdoch, D.A. Hammer, T.P. Anderson); Naval Medical Research Center, Silver Spring, Maryland, USA (P.J. Rozmajzl, A.L. Richards); Duke University Medical Center, Durham, North Carolina, USA (C.W. Woods, L.B. Reller); Durham VA Medical Center, Durham (C.W. Woods)

**Keywords:** Murine typhus, Rickettsia typhi, PCR, Nepal, typhoid, dispatch

## Abstract

Murine typhus was diagnosed by PCR in 50 (7%) of 756 adults with febrile illness seeking treatment at Patan Hospital in Kathmandu, Nepal. Of patients with murine typhus, 64% were women, 86% were residents of Kathmandu, and 90% were unwell during the winter. No characteristics clearly distinguished typhus patients from those with blood culture–positive enteric fever.

In 2001, we found *Salmonella enterica* serotype Typhi and *S. enterica* serotype Paratyphi A to be the most common causes of bloodstream infections among adults with febrile illness who sought treatment at Patan Hospital in Kathmandu, Nepal (*1*). Another important finding was the relatively high percentage of patients (11%) who had immunoglobulin (Ig) M antibodies against *Rickettsia typhi* in peripheral blood. Because most testing was performed on unpaired acute-phase sera, and a high percentage of seropositive results were found in a group of healthy study participants, we were uncertain whether these participants had acute murine typhus or more distant past infection.

Recent studies have shown the value of PCR for diagnosing scrub typhus (*2**–**4*), and a real-time assay for *R. typhi* has recently been validated (*5*). In our study, we tested archived blood samples from our febrile adult cohort (*1*) with this *R. typhi* PCR to better characterize the incidence of murine typhus and to determine whether clinical features could help distinguish murine typhus from enteric (typhoid) fever*.*

## The Study

We studied consecutive adult patients with fever (axillary temperature >38°C; >13 years of age) who sought treatment at Patan Hospital from January 15 through March 15, 2001 (winter) and July 2 through August 10, 2001 (summer), as detailed elsewhere (*1*). The study was approved by the Nepal Health Research Council and the Institutional Review Board of the Centers for Disease Control and Prevention.

Blood from each patient was injected into blood culture bottles and serum samples were tested for *R. typhi* IgM antibodies (INDX Multi-Test Dip-S-Ticks SDLST; Integrated Diagnostics, Inc., Baltimore, MD, USA). In addition, whole blood samples (stored at −80ºC) were tested by real-time PCR for *R. typhi* at the Naval Medical Research Center, Silver Spring, MD (NMRC) and at Canterbury Health Laboratories, Christchurch, New Zealand (CHL). Details of the assay have been described elsewhere (*5*). We extracted DNA from 200 μL of whole blood and used primers and probes specific to a portion of the outer membrane protein B (*ompB*) unique to *R. typhi* to amplify and detect the target sequence in a SmartCycler (Cepheid, Sunnyvale, CA, USA) at NMRC and in a LightCycler (Roche Diagnostics, Mannheim, Germany) thermocycler at CHL. Thermocycling parameters included an initial denaturation step (2 min at 9°C) followed by 45 cycles of denaturation (94°C for 5 s) and annealing/elongation (60°C for 30 s) steps. Positive samples were defined as those that demonstrated fluorescence above background levels. Template-free controls assayed at the same time and under the same conditions as the experimental and positive control samples consistently showed negative results.

In our study, a diagnosis of murine typhus required a positive *R. typhi* PCR result; a diagnosis of enteric fever required a positive blood culture for *S.* Typhi or *S.* Paratyphi. Data from patients with murine typhus were compared with those from patients with enteric fever by using the χ^2^ test or Fisher exact test for dichotomous and ordinal variables, and 2-sided Wilcoxon rank sum test and the Student *t* test for continuous variables. We used multivariable logistic regression analysis to further evaluate variables associated with murine typhus. Murine typhus was the outcome variable in the final model; other variables were those associated with the outcome with p<0.1 on bivariable analysis. Data were analyzed by using STATA version 8.2 (StataCorp, College Station, TX, USA).

We enrolled 876 patients, 370 in winter and 506 in summer. In 323 (37%) patients, a putative diagnosis was established; 117 (13%) patients had positive blood cultures for *S.* Typhi or *S.* Paratyphi A.

Whole blood samples were available for testing from 756 (86%) patients. Of these patients, 85 (11%) had *R. typhi* IgM antibodies detected in acute-phase serum samples and 50 (7%) had positive *R. typhi* PCR results; 11 (13%) of the *R. typhi–*seropositive patients were also PCR positive. Sequencing of amplicons from 5 PCR-positive patients showed 100% similarity with the reference strain of *R. typhi* (GenBank accession no. AE017197). None of the patients with positive PCR results for *R. typhi* had a positive blood culture.

The features of the 50 patients with murine typhus and the 94 patients with enteric fever are presented in the Table; all had negative *R. typhi* PCR results. Sixteen of the murine typhus patients had chest radiographs; 6 (38%) of these patients were reported as having lung infiltrates. Of the 50 patients with murine typhus, 45 were managed as outpatients and 5 were ill enough to be admitted to a hospital. No deaths occurred.

**Table:** Demographic, clinical, and laboratory features of patients with murine typhus and enteric fever, Nepal*

Variable	Murine typhus, n = 50	Enteric fever, n = 94	p value
Demographics			
Age, y, median (range)	28 (15–85)	22 (14–72)	0.0001
Male gender	18 (36)	61 (65)	0.001
Occupation			0.001
Housewife	17 (41)	10 (12)	
Student	7 (17)	36 (42)	
Business person	2 (5)	7 (8)	
Government employee	2 (5)	8 (9)	
Other	13 (32)	24 (28)	
Residence			<0.001
Kathmandu	31 (86)	27 (32)	
Patan	0	28 (33)	
Kathmandu Valley	4 (11)	10 (12)	
Other	1 (3)	20 (24)	
Winter season	45 (90)	27 (29)	<0.001
Admission diagnosis			<0.001
Enteric fever	21 (42)	66 (70)	
Lower respiratory tract infection	13 (26)	3 (3)	
Urinary tract infection	6 (12)	3 (3)	
Upper respiratory tract infection	3 (6)	1 (1)	
Other	7 (14)	21 (22)	
Symptoms			
Cough	33 (66)	30 (33)	<0.001
Shortness of breath	16 (32)	6 (7)	<0.001
Nausea	14 (28)	34 (37)	0.26
Diarrhea	5 (10)	16 (18)	0.24
Abdominal pain	16 (33)	25 (28)	0.58
Headache	41 (82)	78 (86)	0.56
Joint pain	12 (24)	11 (12)	0.07
Duration of symptoms, d, median (range)	5 (1–10)	5 (1–30)	0.23
Examination findings			
Temperature, °C, mean (SD)	38.9 (0.7)	38.8 (0.6)	0.54
Respiratory rate, breaths/min, mean (SD)	26 (9)	21 (5)	0.0002
Heart rate, beats/min, mean (SD)	112 (17)	105 (15)	0.02
Systolic blood pressure, mm Hg, mean (SD)	109 (17)	107 (11)	0.52
Diastolic blood pressure, mm Hg, mean (SD)	70 (10)	70 (8)	0.88
Crackles	13 (26)	8 (9)	0.006
Hepatomegaly	2 (4)	9 (10)	0.22
Splenomegaly	2 (4)	12 (13)	0.09
Rash	0	1 (1)	0.46
Laboratory findings			
Hematocrit, %, mean (SD)	39 (6)	39 (5)	0.70
Leukocyte count, cells × 10^9^/L, median (IQR)	8.9 (6.2–11.1)	5.8 (4.8–7.6)	<0.001
Neutrophils, %, median (IQR)	83 (74–87)	68 (60–73)	<0.001
Lymphocytes, %, median (IQR)	13 (9–22)	27 (20–35)	<0.001
Monocytes, %, median (IQR)	2.5 (1–5)	2 (0–4)	0.57
Eosinophils, %, median (IQR)	0.5 (0–2)	0 (0–2)	0.80

*Data are no. (%) unless otherwise stated. SD, standard deviation; IQR, interquartile range.

After logistic regression analysis, only 3 variables were significantly associated with murine typhus compared with enteric fever. These variables were age (for each increase by 1 year) (odds ratio [OR) 1.07, 95% confidence interval [CI] 1.00–1.16, p = 0.05); Kathmandu residence (OR 14.37, 95% CI 1.07–193.39, p = 0.05); and winter season (OR 28.93, 95% CI 2.47–338.93, p = 0.007).

## Conclusions

We detected *R. typhi* DNA in blood from 7% of the febrile adult study population from urban Nepal. This finding is likely to be an underestimate of the actual extent of rickettsial disease because of the small volume of blood tested in each PCR and possible sample deterioration during transport and storage (between sample collection and testing). Although PCR has yet to be extensively evaluated for the diagnosis of murine typhus (*5**–**8*), a sizeable body of evidence supports the high sensitivity and specificity of PCR for the diagnosis of rickettsial diseases (*2**,**4**,**9**,**10*). The real-time PCR used in our study has a high analytical sensitivity and specificity for *R. typhi* (*5*), and sequencing of amplicons from our patients further supports the specificity of the assay. In addition, none of our patients with murine typhus had positive blood cultures.

The results of this and other (*11**,**12*) studies indicate that murine typhus is an important endemic infection in Nepal. Although our study did not extend throughout the full year, murine typhus was more common in winter than in summer. This finding contrasts with the summer–autumn predominance reported in other regions (*13*,*14*). We also noted a clear predominance of cases from Kathmandu and none from the Patan side of the city, despite the fact that the latter is the main catchment area for Patan Hospital. It is possible that an outbreak of murine typhus occurred in Kathmandu during the winter of 2001, and epidemiologic studies are needed to clarify whether there is a focus of murine typhus activity in Kathmandu. In Nepal, enteric fever is one of the most common causes of febrile illness (*1**,**15*). We identified no reliable clinical marker to distinguish murine typhus from enteric fever, and the classic clinical triad of rickettsial diseases (headache, fever, and rash) was not detected in any of our patients. Murine typhus should be considered as an alternative diagnosis in patients with suspected enteric fever in Nepal. This diagnosis is especially important given that first-line antimicrobial drug therapy is different for the 2 diseases.

Our study highlights the importance of rickettsial infections as a cause of febrile illness in Kathmandu. Further epidemiologic and ecologic studies are needed to better clarify the features of murine typhus in Nepal.
